# Dimensionality and predictive validity of the HAM-Nat, a test of natural sciences for medical school admission

**DOI:** 10.1186/1472-6920-11-83

**Published:** 2011-10-14

**Authors:** Johanna C Hissbach, Dietrich Klusmann, Wolfgang Hampe

**Affiliations:** 1 Department of Biochemistry and Molecular Cell Biology, Center for Experimental Medicine, University Medical Center Hamburg-Eppendorf, Martinistraße 52, 20246 Hamburg, Germany; 2 Department of Medical Psychology, Center for Psychosocial Medicine, University Medical Center Hamburg-Eppendorf, Martinistraße 52, 20246 Hamburg, Germany

## Abstract

**Background:**

Knowledge in natural sciences generally predicts study performance in the first two years of the medical curriculum. In order to reduce delay and dropout in the preclinical years, Hamburg Medical School decided to develop a natural science test (HAM-Nat) for student selection. In the present study, two different approaches to scale construction are presented: a unidimensional scale and a scale composed of three subject specific dimensions. Their psychometric properties and relations to academic success are compared.

**Methods:**

334 first year medical students of the 2006 cohort responded to 52 multiple choice items from biology, physics, and chemistry. For the construction of scales we generated two random subsamples, one for development and one for validation. In the development sample, unidimensional item sets were extracted from the item pool by means of weighted least squares (WLS) factor analysis, and subsequently fitted to the Rasch model. In the validation sample, the scales were subjected to confirmatory factor analysis and, again, Rasch modelling. The outcome measure was academic success after two years.

**Results:**

Although the correlational structure within the item set is weak, a unidimensional scale could be fitted to the Rasch model. However, psychometric properties of this scale deteriorated in the validation sample. A model with three highly correlated subject specific factors performed better. All summary scales predicted academic success with an odds ratio of about 2.0. Prediction was independent of high school grades and there was a slight tendency for prediction to be better in females than in males.

**Conclusions:**

A model separating biology, physics, and chemistry into different Rasch scales seems to be more suitable for item bank development than a unidimensional model, even when these scales are highly correlated and enter into a global score. When such a combination scale is used to select the upper quartile of applicants, the proportion of successful completion of the curriculum after two years is expected to rise substantially.

## Background

The development of student admission procedures is of major interest to medical schools worldwide. Poor fit of students' interests and talents with the course may lead to dropout, delay in study progress and low grades [1]. Given the cost of medical degree programmes, the responsibility of medical schools is to graduate competent physicians [2], and given the impact poor academic success can have on self-confidence and self-esteem of students [3] valid measures should be sought to identify prospective successful students.

A review of the literature [4] concluded that pre-university academic achievement predicted academic success in undergraduate training with an average effect size of d = .30 and postgraduate training with an average effect size of d = .14. Prior academic performance is commonly assessed by grade point average (GPA) scores (USA), or A-levels (UK). In a large-scale longitudinal study, high school or undergraduate grade point average (uGPA) was related to cumulative GPA in the first two years of medical school with a validity coefficient of r = .40 (r = .54 if corrected for range restriction) [5]. GPA is easily available, cost-efficient, comparatively reliable and valid [6]. In American studies the predictive power regularly exceeds that of sophisticated assessment procedures like the SAT2, known as the "Scholastic Assessment Test" or "Scholastic Aptitude Test" and the American College Testing Program (ACT) [7,8]. Similarly, in a German meta-analysis, the German high school GPA, which is mandatory for student selection, showed the strongest association with academic success in the first two years of medical school (r = .58 with examination marks, corrected for reliability of the criterion and restricted range) [9]. However, the German high school GPA is not fully comparable across German states, which vary in curricula and evaluative standards [9]. As this variation may lead to inequity, some German medical schools use additional measures for student selection such as the "Test for Medical Studies" (TMS) [10]. In the USA the "Medical College Admission Test" (MCAT) fulfils a similar role and in the UK it is the "BioMedical Admission Test" (BMAT). The scientific knowledge section of the BMAT [11,12] and the biological sciences subtest in the MCAT [13] were the best predictors of examination marks in the preclinical years. A dramatic effect of student selection was reported by a study from the Medical University of Graz, Austria [14]. Until 2005 in Austria every applicant for the study of medicine was admitted without selection. With this free admission policy only 22.8% of the 2004 cohort completed the first part of the study programme within the scheduled time of 1 year. In the year of 2005 a selection procedure was introduced, and subsequently the proportion of successful completions after one year rose threefold to 91.9%. The selection procedure required applicants to complete a probationary semester concluded by 2 days of examinations. In subsequent cohorts, a less demanding procedure consisting of a single natural science knowledge test was used for selection, but the rate of success after one year continued to range at high levels (85.9% in the 2006, and 75.6% in the 2007 cohort).

In 2006 Hamburg Medical School decided to make admission conditional on the level of knowledge in natural sciences. The first version of the Hamburg Assessment Test for Medicine, Natural Sciences (HAM-Nat) [15] was developed in 2006. It consists of 52 multiple-choice items from physics (15 items), biology (14 items), and chemistry (20 items). Three easy items with mathematical content were included in the test as warm-up items.

The test's objectives are (a) to identify applicants with good chances to succeed in the first two years and (b) to inform prospective applicants about what to expect in medical courses by offering access to typical questions on the school's website (http://www.uke.de/studierende).

As high stakes application tests like the HAM-Nat cannot be used repeatedly for every new cohort of applicants, fresh items are needed every year. The long-term aim of our project is to accumulate a database of tested items that fit a common scale and thus are interchangeable with respect to that scale. Item response theory (IRT) is the most feasible framework for this goal. It facilitates test equating through a simple linear transformation, and its rich description of item performance provides guidance for the writing of new items. IRT models have been used in the context of medical education, e.g. for achievement testing [16], licensing examinations [17], or interview procedures [18]. For an introduction and a comparison of methods within the different test theoretical frameworks (classical test theory vs. IRT) see DeChamplain [19], and for a controversial discussion see Downing [20] and Burton [21].

For this study the freshmen of the 2006 cohort took the HAM-Nat test after having been admitted without the test. For construct validation of the HAM-Nat we explore different ways to conceive of our measure: (a) to take natural science knowledge as a single unified dimension of knowledge, and (b) to differentiate three dimensions according to the field of knowledge: physics, chemistry, and biology. We will investigate how well these scales fit the Rasch model and how they perform in predicting academic success. Finally, we will decide which path to follow in construction of an item bank.

## Methods

### Item response theory

Item response theory (IRT) refers to a family of models for the analysis of measurement scales [22-25]. Among the advantages over classical test theory are testability of model fit, extensive diagnostics for the performance of individual items and persons, and a straightforward procedure for test equating. A mandatory requirement for IRT modeling is unidimensionality of the latent variable. Moreover, at a fixed trait level, item correlations should solely depend on their relations to the latent trait [26]. This is the local independence condition, demanding residual correlations to be zero. Since local independence in this strict sense is hard to achieve it is widely agreed that the dominant factor must be just strong enough to ensure that trait level estimates are unaffected by smaller specific factors [25] and other causes of local dependence. One source of local dependence is of course multidimensionality, but there may be other reasons like similarity in item response format, overlapping content, differential exposure to educational experiences, or fatigue of testees (for an overview see Yen, 1993 [27]). Violation of the local independence assumption results in biased parameter estimations, particularly inflation of reliability coefficients [28].

The basic IRT model is the Rasch model with only one parameter to be estimated: item difficulty (1PL model). In this model the probability of a correct answer solely depends on the ability of the person and the difficulty of the item. More complex models include the estimation of item discrimination and guessing parameters (2PL and 3PL models). Only in the 1PL model the conditions of fundamental measurement as defined by conjoint measurement theory are met [29], and interval quality of the trait scores is guaranteed. Therefore, some researchers insist on the pursuit of Rasch scale properties, even at the price of severe item selection.

Persons with high scores on the latent trait (e.g. high ability) should have a high probability to answer items correctly. Deviations from this expectation are expressed by two misfit coefficients for each item: the infit- and the outfit-statistics. They are based on the mean square of residuals with an expected value of 1. A high value indicates an irregular response pattern, e.g. persons with high abilities giving wrong answers and persons with low abilities giving the right answer. A low value indicates a response pattern which is too predictable, and therefore not informative [30].

### Data collection

Participants of the study had qualified for Hamburg Medical School by different criteria: excellent GPA scores (70%), waiting list (20%), foreign students' quota (8%), as well as several cases of hardship, and lawsuit for admission.

Of the 452 enrolled students, 336 agreed to participate in the study. The HAM-Nat was administered in a 1.5-hour session during the first week of the first term in October 2006. Two years later, the participants' academic success was assessed using a student administration database. After exclusion of two subjects, who could not be matched with administration data, the sample finally contained 334 respondents (74% of the cohort). The mean age was 22.2 years (SD = 4.22, range 18.5 - 50.7) and the mean high school GPA was 1.76 (SD = .55, range 1.0 - 3.7). The proportion of female students was 64%. Data were made anonymous and all participants gave written informed consent. Ethical approval has been waived, but a statement of ethical considerations confirming that ethical principles have been adhered to is provided (see additional file 1: Statement of ethical considerations).

### Measures

The 52 multiple-choice items of the HAM-Nat are intended to assess high school level natural science knowledge, relevant to the medical curriculum. They were devised by high school teachers and university lecturers. Each item presents one correct answer and four distractors.

Example:

Oxidation of an aldehyde yields...

A) an ester. B) a ketone. C) a carboxylic acid. D) an alcohol. E) an alkene.

In a preliminary study the HAM-Nat score was only weakly related to high school GPA (r = -.21) [15].

We used the pool of 52 HAM-Nat items to explore alternative approaches to item bank development. The first approach was to combine items into a single unidimensional Rasch scale for natural science knowledge (HAM-Nat-uni), regardless of subject area. The second approach was to generate three Rasch scales for biology, physics, and chemistry and combine these subscales into one (HAM-Nat-BPC). The predictive power of the HAM-Nat was assessed with a dichotomous outcome measure for academic success: qualification for the first medical exam after two years (*SUCCESS*).

### Data analysis

Exploratory weighted least squares (WLS) factor analysis (Mplus software [31]) was used to create a unidimensional scale for natural science knowledge (HAM-Nat-uni) by selecting items with high loadings in the single factor solution. In contrast to conventional factor analysis, WLS factor analysis uses tetrachoric correlations instead of product-moment-correlations to avoid the confounding influence of item skewness [32,33]. The three items sets specific to the subject areas biology, physics, and chemistry were analysed in the same way. All scales were checked for locally dependent item sets and item pairs with residual correlations r_res_>|.20| were examined [34]. If content overlap or any other irregularity were detected in a set of items, a single representative item was chosen as a replacement. Items with residual correlations exceeding |.25| were excluded regardless of content. Finally, the resulting item set was analysed to determine its conformity to the Rasch model.

We only estimated the 1PL (Rasch) model because the small sample size precluded estimation of 2PL and 3PL models. Model data fit was assessed with the program Winsteps [35] which uses joint maximum likelihood estimation to estimate item and person parameters simultaneously. All items with significant misfit to the model (standardized mean square values ZSTD > |1.96|) were excluded and the model was reestimated until all items fit to the scale.

Generally, scales which are fitted to a specific sample are not guaranteed to maintain their properties in another sample. In order to control this effect of overfitting, we split the data set of 334 students into two random subsamples A and B. Sample A, designated as the development sample, was used to construct various scales from the item pool. The validity of the dimensional models was checked by confirmatory WLS factor analysis (factor variance fixed at one) in validation sample B. Rasch analysis was repeated to assess how well scale properties had survived the transfer to another sample.

We used the dichotomous outcome criterion *SUCCESS *(qualification for the first medical exam after two years) as target variable with logistic regression analysis [36], executed by SPSS 17 and 19 for Windows [37].

## Results

### Description of samples

After two years, 234 of 334 students (70.1%) had completed the regular curriculum. There were no statistically significant differences between genders and between the two random subsamples A and B (Table 1).

**Table 1:** Description of samples

	Total sample (334)	Sample A (167)	Sample B (167)
Abiturnote^1 ^(Mean)	1.76	1.72	1.80
Female (%)	64.1	64.7	63.5
Correct answers in HAM-Nat-52, total sample (%)	49.0	48.8	49.3
Correct answers in HAM-Nat-52, females (%)	48.1	47.9	48.3
Correct answers in HAM-Nat-52, males (%)	50.7	50.5	51.3
Success after 2 years, total sample (%)	70.1	71.9	68.3
Success after 2 years, females (%)	72.4	72.2	72.6
Success after 2 years, males (%)	65.8	71.2	60.0

^1^The Abiturnote is the German equivalent to high school GPA, the meaning of scale values is reverted: 1.0 is the best and 4.0 the lowest possible score.

### Rasch scale for knowledge in natural sciences as a single dimension

**Figure 1:**
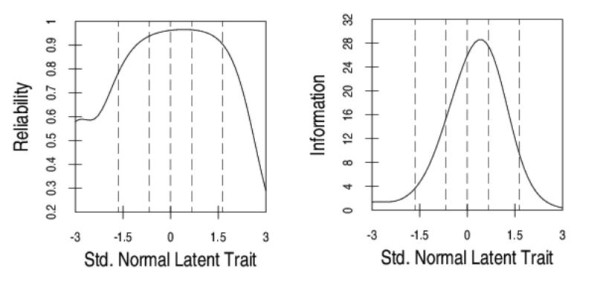
**Reliability curve and item information curve for HAM-Nat-uni**.

In the pool of 52 HAM-Nat items the mean tetrachoric inter-item correlation was r = .20, and the ratio of the first to the second eigenvalue was 2.9 for development sample A. The single factor solution was used to select a set of 33 items with factor loadings >0.4 (range: 0.42 - 0.76) for a unidimensional scale. The matrix of residual correlations produced by this selected item set contained a proportion of 5.1% coefficients larger than r_res _= |.20|. Analysis of item content yielded no apparent reasons for these residual correlations. Since no residual correlation exceeded r_res _= |.25|, no item was excluded. All of the 33 items fitted to the Rasch model with standardized infit and outfit mean square statistics ZSTD < |1.96|. This scale of 33 items (12 physics, 14 chemistry, 6 biology, 1 mathematics) selected for unidimensionality was designated HAM-Nat-uni. The scale's internal consistency was .84 (Table 2). In IRT, a scale's reliability (or in IRT terminology "precision") varies across the latent trait. In the decisive region around a latent trait value of 0.5 (the hypothetical cut-off score for the top achieving 25%) reliability was close to .90, and item information was at its maximum (Figure 1). Therefore, precision is maximal where it is needed most: in the area where decisions about admission or rejection are made.

**Table 2:** Scales constructed from the HAM-Nat item pool

Scale	Description	Items	**Internal consistency**^**1 **^**sample A**	**Internal consistency**^**1 **^**sample B**
HAM-Nat-raw	Sum of the total item pool	52	.87	.85
HAM-Nat-uni	Unidimensional scale	33	.84	.81
HAM-Nat-biology	Subject-specific scale	11	.75	.73
HAM-Nat-physics	Subject-specific scale	13	.75	.58
HAM-Nat-chemistry	Subject-specific scale	14	.70	.69
HAM-Nat-BPC	Unweighted sum of subject-specific scales	38	.85	.81
HAM-Nat-BPCw	Weighted sum of subject-specific scales with weights derived from sample A (0.45, 0.43, 0.15).	38	.85	.81
HAM-Nat-corrmax	Scale composed of the 33 items, most highly correlated with *SUCCESS *in sample A	33	.83	.79

^1 ^Cronbach's alpha

**Figure 2:**
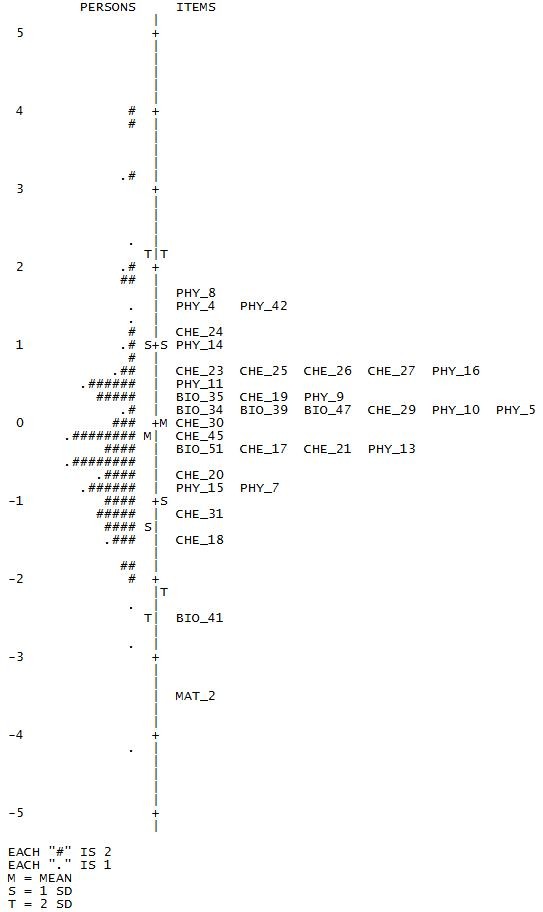
**Item-Person-Map, HAM-Nat-uni, Sample A**.

The item-person-map locates item difficulties and person abilities on a common logit-scale (Figure 2). A person with an ability score equal to an item's difficulty has a chance of 50% to answer that item correctly. As item and person parameters are estimated separately and the mean item difficulty is set to 0 by default, a mean person ability of -.20 indicates that most respondents solved less than half of the items. If the goal had been optimal discrimination in the middle range, this test would be slightly too difficult for this sample. However, the test will typically be used for selection at the upper end of the sample. Therefore, it should discriminate best in the decisive region and even more difficult items should be added in order to maximize test information.

To cross-validate this approach, the same 33 items were subjected to confirmatory factor analysis in validation sample B. Nearly half of the loadings on the single factor dropped below 0.4 and the proportion of residual correlations r_res_>|.20| increased from 5.1% to 12.0%. The one factor solution found in sample A was not sufficient to explain the correlation matrix in sample B (CFI = .89; TLI = .90; RMSEA = .046). Of the 33 items, 8 showed poor fit to the Rasch model (ZSTD > |1.96|).

In conclusion, the discrepancy of eigenvalues between the first and the second factor suggest a unidimensional structure, but as correlations are generally weak, this factor is weak, too. Even after severe item selection for unidimensionality in test sample A the resulting scale did not conform to one single dimension when its definition was applied to validation sample B. Likewise the scale reasonably satisfied the fit criteria of the Rasch model in development sample A but not in validation sample B.

### Rasch scales for physics, chemistry, and biology

In the 3-dimensional approach we separated the physics, chemistry, and biology items into individual scales and subjected each scale to a WLS factor analysis with a one-factor-solution. A total of four items were excluded due to factor loadings <.40 on their respective scales. The proportion of residual correlations r_res_>|.20| were 1.8% in the biology scale, 3.8% in the physics scale, and 6.6% in the chemistry scale. After removal of two items due to r_res_>|.25|, one from physics and one from biology, these scales showed sufficient fit to the Rasch model (all infit and outfit ZSTD < |1.96|). The chemistry scale was reestimated after the exclusion of one underfitting and three overfitting items until sufficient model-data fit was obtained. An example: Two overfitting items presented the same computation problem of molar mass from different angles - one was kept, one was excluded.

To validate the three-dimensional model, 3-factor confirmatory WLS factor analysis was executed in validation sample B. The 3-factor model showed good fit to the data (CFI = .97; TLI = .97; RMSEA = .023) when factors were allowed to correlate (r_(Physics, Chemistry) _= .76; r_(Physics, Biology) _= .57; r_(Chemistry, Biology) _= .51). These are high correlations which indicate that the item set is not really three-dimensional. Taken to sample B the three item sets defined by the 3-factor model in sample A fit well to a Rasch model with only one item of each scale exceeding the range of acceptable misfit.

Then how should the subject-specific scales be combined? One possibility is to simply add up the three subject scores to one global score. In this case biology, physics, and chemistry are given the same weight to obtain the scale (see HAM-Nat-BPC in Table 2). In Rasch analysis, this scale showed sufficient fit to the model. In the validation sample, all items except one biology item had infit and outfit ZSTD values <|1.96|. This would be the optimal combination if all of the three subject-specific scales were independently related to academic success with the same strength. We tested the latter assumption with logistic regression analysis in sample A. The regression coefficients for biology and physics were similar (β = .47 and .43) and significant or close to significance (p = .024 and .069); chemistry, however, did not perform as well (β = .15, p = .522). With these coefficients, we computed a weighted summary scale, HAM-Nat-BPCw, that will be compared with its unweighted counterpart in terms of predictive power.

### Scale selection by item-criterion correlations

Academic success will always be the predictive target variable for the HAM-Nat. Therefore, it may be promising to exploit predictive information for scale construction. To examine this approach the 33 items correlating highest with *SUCCESS *in sample A were combined into a scale named HAM-Nat-corrmax (Table 2). This procedure may be termed pragmatic or opportunistic item selection. We fixed the number of items to be selected by the magnitude of their correlation with the criterion at 33 items to be able to compare the resulting scale (HAM-Nat-corrmax) with another 33-item scale, the HAM-Nat-uni - which had been constructed on the basis of the Rasch model. The four different subject areas were represented in the HAM-Nat-corrmax with 12 items from physics, 11 from chemistry, and 9 from biology. In the HAM-Nat-uni the corresponding proportions were 12 physics, 14 chemistry, 6 biology, 1 mathematics.

### Predictive power of the different scales

We compare the predictive power of eight different ways to combine item information into scales (Table 3). Target variable is the dichotomous criterion *SUCCESS*. All scales in Table 3 are fitted to development sample A. Therefore, all scales should have better psychometric properties in development sample A as compared to validation sample B and therefore should work better in sample A than in sample B as predictors of *SUCCESS*.

**Table 3:** Prediction of academic success in development sample A and validation sample B; Target variable: SUCCESS (qualified for the first step of the medical exam after 2 years)

		Development Sample A				Validation Sample B				Total sample			
		
	**Predictor variable **^**1**^	odds ratio	p	95%-CI		odds ratio	p	95%-CI		odds ratio	p	95%-CI	
1	HAM-Nat-raw	2.23	.001	1.41	3.54	1.80	.003	1.24	2.62	1.97	.000	1.47	2.63
2	HAM-Nat-uni	2.05	.001	1.33	3.17	1.70	.005	1.18	2.45	1.84	.000	1.39	2.43
3	HAM-Nat-biology	1.76	.002	1.22	2.53	1.65	.004	1.17	2.33	1.70	.000	1.32	2.18
4	HAM-Nat-physics	1.77	.005	1.19	2.65	1.54	.021	1.07	2.23	1.65	.000	1.26	2.16
5	HAM-Nat-chemistry	1.59	.019	1.08	2.35	1.40	.056	0.99	1.97	1.48	.003	1.15	1.91
6	HAM-Nat-BPC	2.23	.001	1.40	3.53	1.78	.003	1.22	2.59	1.95	.000	1.46	2.61
7	HAM-Nat-BPCw	2.25	.000	1.44	3.53	1.85	.002	1.26	2.72	2.01	.000	1.50	2.69
8	HAM-Nat-corrmax	3.02	.000	1.83	5.00	1.67	.005	1.16	2.40	2.12	.000	1.58	2.84

Logistic regression

^1 ^All predictor variables are z-transformed. Two persons differing in their scores by 1 standard deviation will differ in their odds to achieve success after two years by the odds ratio. For scale definitions see Table 2

In fact, odds ratios are consistently higher in development sample A than in validation sample B by a factor of 1.15 (except for HAM-NAT-corrmax, the scale which exploits predictive information). Is this an effect of psychometric deterioration after transfer of scale definitions from sample A to sample B? The raw score HAM-NAT-raw is unaffected by any method of item selection. Therefore, the drop in odds ratios cannot be attributed to item selection and overfit-related deterioration, but most probably reflects chance differences between the randomly generated subsamples A and B. From this we conclude that the transfer of scale definitions from sample A to sample B did not influence predictive power substantially.

The odds ratio for the scale HAM-Nat-uni is only slightly lower than the odds ratio for the raw scale HAM-Nat-raw, even though 36% of items were excluded. The three short subject-specific scales produce low but significant or close to significant odds ratios, with chemistry scraping at the .05 level. HAM-Nat-BPC, the unweighted linear combination of subject-specific scales, seems to predict *SUCCESS *slightly better than the unidimensional scale. Weighting does not significantly improve the predictive power of the HAM-NAT-BPC-scale (odds ratio is 1.85 in the weighted version of the scale vs. 1.78 in the unweighted version, sample B).

The linear combination of biology, physics, and chemistry weighted by .47, .45, and .15 is named HAM-Nat-BPCw. When taken from development sample A to validation sample B, predictive power is expected to decline due to overfit of the regression weights and in fact the odds ratio declines (Table 3). However, the drop from 2.25 to 1.85 seems to be in line with chance differences in predictive potency between samples.

Overfit is clearly a problem of the scale HAM-NAT-corrmax. In development sample A, this scale produced a high odds ratio of 3.02, but transferred to validation sample B the value dropped to 1.67. This drop demonstrates the effect of overfitting caused by random associations exploited in the development sample, which disappeared in the validation sample as they regressed to the mean. The odds ratio of 1.67 is slightly lower than the one obtained with HAM-Nat-uni. Thus, a pragmatic (or opportunistic) stance, just demanding validity for a specific criterion, did not pay. The Rasch scale HAM-Nat-uni, performing as well, offers the advantage of measuring a latent variable - regardless of the criterion used for validity assessment - and of the total score being interval-scaled.

### Estimated gain in success rate depending on proportion of selected applicants

What do we gain by using the HAM-Nat? An extrapolation of results for different proportions of selected applicants (no selection at all, proportions of ¾, of ½, and of ¼) gave following results: The expected success rate increases from 70.1% to values between 74.1% and 85.5%, depending on the selected proportion and the specific predictor used (Table 4). With the HAM-Nat-BPC and a selected proportion of ¼, an absolute gain of 13.0% in the proportion of successful applicants is expected and this corresponds to an effect size of h = .32 (computed by arcsin-transformation of proportions; h is equivalent to d) [38] (pp. 179).

**Table 4:** Expected proportions of academic success after 2 years depending on proportion of selected applicants and information used for selection.

	Proportion of students expected to be successful after 2 years if cohort is initially selected by...		
**Selected proportion**	**HAM-Nat-raw**	**HAM-Nat-uni**	**HAM-Nat-BPC**
100%	70.1	70.1	70.1
75%	75.4	75.2	74.1
50%	81.6	81.0	79.6
25%	84.6	85.5	83.1

### HAM-Nat and GPA

The German equivalent to the GPA (Abiturnote) is mandatory for student selection. Does using the HAM-Nat provide predictive gain in addition to GPA? We used the combined scale HAM-Nat-BPC to assess its independent predictive contribution (incremental validity), after GPA had been taken into account by statistical control in a logistic regression. Taken alone, the scale HAM-Nat-BPC predicts academic success (Table 3). When GPA is included in the regression equation as an additional predictor, the relation of the scale HAM-Nat-BPC to academic success remains virtually unchanged (compare Table 3 and Table 5). Thus, the HAM-Nat BPC scale provides independent predictive information for academic success, in addition to the information provided by the GPA.

**Table 5:** The contribution of HAM-Nat-BPC in the prediction of academic success (1) when GPA and (2) when gender is included

		Total sample			
		
Model	Predictor variable	odds ratio	p	95% CI	
1	HAM-Nat-BPC and GPA				
	HAM-Nat-BPC	1.91	.000	1.40	2.60
	GPA	1.62	.000	1.26	2.08
	HAM-Nat-BPC × GPA	1.07	.630	0.82	1.40
2	HAM-Nat-BPC and gender				
	HAM-Nat-BPC	2.50	.000	1.40	2.60
	Gender*	1.62	.071	.96	2.73
	HAM-Nat-BPC x Gender	1.55	.143	.86	2.78

Target variable: SUCCESS (qualified for the first step of the medical exam after 2 years). Logistic regression. * male=0, female=1, the odds of success are greater in females than in males by the ratio 1.62.

### Gender

**Figure 3:**
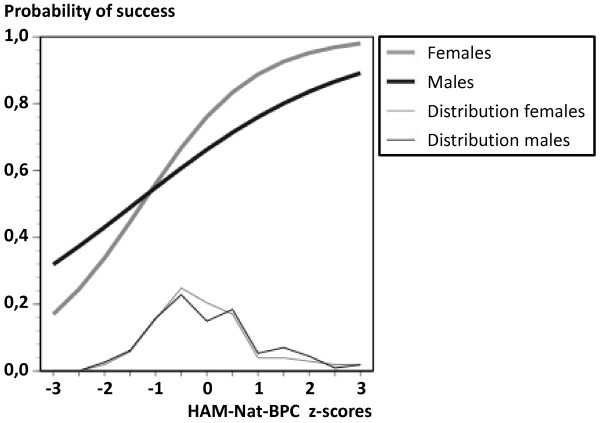
**Probability of success as predicted by HAM-Nat-BPC, split up by gender, logistic regression, total sample**.

Success after two years tended to be slightly higher in females than in males (p(t) = .163) while HAM-Nat scores tended to be slightly higher in males than in females (p(t) = .207). In the logistic regression equation (Table 5) these contrary tendencies result in a suppressor effect which slightly elevates the predictive power of the HAM-Nat-BPC (odds ratio of 1.95 to 2.50) and drives the gender effect close to significance (p = .071). Visual inspection of Figure 3 suggests that the curves relating the HAM-Nat-BPC to success might be differentially shaped for males and females, the steeper curve indicating a better prediction of academic success in females than in males. However, the effect of the interaction "gender by HAM-Nat-BPC" is not significant (p = .143, Table 5).

### Predictive power in the upper quartile of the HAM-Nat

**Figure 4:**
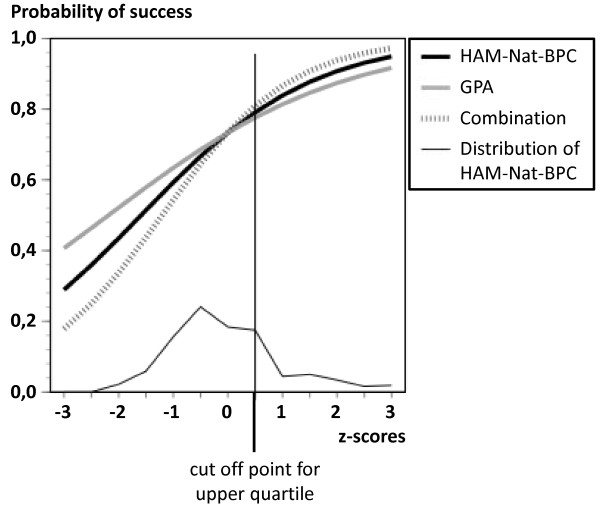
**Probability of success as predicted by HAM-Nat-BPC when GPA is controlled, GPA when HAM-Nat-BPC is controlled, and an optimal linear combination of both variables, logistic regression, total sample**.

If the HAM-Nat had been used with a policy of accepting only the 25% of applicants with the highest scores, every applicant to the left of the cut-off point in Figure 4 would have been rejected. The variation of the predictor (HAM-Nat-BPC) and the outcome (academic success) would both have been attenuated, and the error term for the estimation of the B-weights in logistic regression enlarged. Our data allow a preview of this effect: In the upper quartile of HAM-Nat-BPC the variance of HAM-Nat scores decline from 1.0 to .31 and the variance of *SUCCESS *declines from .29 to .18. In a logistic regression, using only the 84 cases located in the upper quartile of the BPC, the shapes of the curves shown in Figure 4 are fairly preserved. However, the standard error of the B-weight for BPC rises fourfold from .157 to .623, and the probability of the Wald-statistic rises from p = .000 to p = .269.

## Discussion

We explored the psychometric properties of various scales derived from a set of 52 natural science items and assessed their predictive power. Two options have been compared (1) to conceive of natural science knowledge as a single dimension and (2) to separate natural science knowledge into the dimensions physics, biology and chemistry.

### Psychometric properties

The second model separating scales for physics, chemistry, and biology items fitted the inter-item correlational structure well and this was reproduced in the validation sample. Moreover, every single subject-specific scale fitted the Rasch model. However, the three scales were highly correlated and this reflects a property of our data set: weak inter-item correlations which neither support a clear unidimensional structure nor a structure of independent components.

In this situation we have to choose pragmatically what is most convenient for the development of an item bank. The single dimension model is parsimonious and it directly relates to the goal of producing one single score for each applicant. However, its departure from unidimensionality in the validation sample will most probably continue to cause insufficient fit to the Rasch model. Therefore, a dimensional model seems more auspicious for future development, even though scale values are combined in the end and weights do not seem to matter much for prediction (see [39] for a discussion of omitted regression weights). Furthermore, a dimensional approach has the benefit of detecting weak components such as the chemistry scale, which does not contribute much to prediction and it gives control over the mix of subject-specific content in future tests.

### Benefit of the IRT model

IRT analysis provides diagnostics for item selection that are not available in classical test theory. It attracts attention to redundant items through its fit statistics. Redundant items seem to perform well due to their high correlation with the scale, even though they do not contribute new information. This was the case, e.g. for chemistry items covering the topic of molar mass. Identification and exclusion of such items helps to avoid inflated estimations of reliabiliy and to make trait measures independent of the specific items used. A second benefit of the IRT approach is the rich information on item performance, e.g. the item characteristic curve and the item-person map which facilitates item selection and directs attention to problems with content and wording. Thirdly, new test forms can be linked simply by a linear transformation based on parameters estimated from a set of overlapping items.

### Predictive power

All summary scales derived from the HAM-Nat item pool predicted academic performance during the first two years of the medical curriculum. No scale definition was clearly superior to the other. Prediction in the validation sample was generally less powerful than in the development sample. This seems to be largely attributable to chance differences between samples, and apart from this the transfer of scale definitions from sample A to sample B did not seem to influence predictive power. However, there is one exception: With item selection by magnitude of item/criterion correlation predictive power dropped substantially due to regression to the mean. The chemistry scale contributed less to prediction than physics or biology, but weighting the scales according to their B-weights did not have a discernible effect on prediction.

The HAM-Nat BPC scale provides independent predictive information for academic success in addition to GPA. Thus, using the HAM-Nat for selection in addition to GPA would increase expected study success and it would offer a second chance to those who otherwise would have been rejected due to a low GPA - of course, the reverse also holds: high GPA-scores no longer guarantee admittance.

With a selected proportion of 25% (using the scale HAM-Nat-BPC) the net gain of successfully completed study episodes over the base rate is expected to rise from 70.1% to 83.1%. In future scenarios, when 200 from 800 applicants are selected by HAM-Nat scores, this amounts to a gain of 26 students not dropping out or delaying. Considering the cost which is imposed on the university and on individual students by dropout and delay, this gain seems to warrant the expenditure of time and energy demanded from applicants and test developers.

When the HAM-Nat is used for selection, academic success data will only be available from the selected group. Will it be possible to estimate the selective power of the HAM-Nat from this group alone? Technically, this is related to the question of whether the logistic regression curve estimated from the upper quartile of our sample (corresponding to a selected proportion of 25%) extends to the lower quartile in the same way as the curve estimated with the full sample information does. While this seems to be the case, the beta coefficient grows extremely unstable and despite its magnitude the effect fails significance. This should be taken into account when the measure's predictive power is assessed with only the selected group as the data base. In combination with GPA the HAM-Nat improved prediction of success by a margin which was of roughly the same size as the contribution provided by GPA alone.

### Gender

Gender differences in the success rate and in HAM-Nat scores were not significant. However, in a logistic regression model including not only the main effects for HAM-Nat scores and gender but also their interaction, the main effect for gender closely failed the .05 significance level. This tendency suggests that female as opposed to male gender might be related to academic success in medical school in an intricate way. The slope of the predictive curve seems to be steeper for females than for males suggesting a stronger predictive relation in females than in males. However, this is a non-significant tendency which would normally not deserve mentioning, but in the case of gender a sensitive issue might be touched. In Graz (Austria) the gender differences in the natural science knowledge test attracted a lot of public attention (e.g. [40]). Gender neutrality would be violated if HAM-Nat scores were substantially more predictive in one gender than in the other.

### Limitations

Our sample of participants who were already admitted deviates from a sample of real applicants mainly in three respects: (1) Participants were probably not as highly motivated as they would have been if admission had been at stake. (2) They had no opportunity to prepare for the test. (3) Not all participants would have needed the test because they would have been admitted for other reasons. Conclusions based on our sample are limited by these deviations from representativeness. On the other hand, a dry run such as this provides information about predictive validity with outcome data from *all *applicants including those who would have been rejected in a real selection procedure - an opportunity that is rare in academic testing.

## Conclusions

Gender specific differential item functioning and differences in predictive power deserve further attention. Adding more difficult items to the HAM-Nat would shift the region of maximum reliability towards a 25% cut-off score. However, with such a shift the problem of guessing is expected to aggravate which might reduce reliability. In theory, guessing can be included into an IRT model, and in later studies with large numbers of applicants this might be a realistic option. The next step will be to establish an item bank partitioned into three subject specific item collections with Rasch scale quality that will be combined into a global scale of natural science knowledge.

## Competing interests

The authors declare that they have no competing interests.

## Authors' contributions

JH and DK performed the statistical analysis and drafted the manuscript. WH conceived of the study, and participated in its design and coordination and helped to draft the manuscript. All authors read and approved the final manuscript.

## Pre-publication history

The pre-publication history for this paper can be accessed here:

http://www.biomedcentral.com/1472-6920/11/83/prepub

## Supplementary Material

Additional file 1 **Statement of ethical considerations**. Ethics-Statement f. Prof. Hampe.pdf Click here for file
