# Supplementary material for: Dimensionality and predictive validity of the HAM-Nat, a test of natural sciences for medical school admission
Source: BMC Med Educ. 2011 Oct 14;11:83. doi: 10.1186/1472-6920-11-83 (PMC3261109; doi:10.1186/1472-6920-11-83)
Supplement: Additional file 1 — Statement of ethical considerations. Ethics-Statement f. Prof. Hampe.pdf [file 1472-6920-11-83-S1.PDF]

Universitätsklinikum Hamburg-Eppendorf

Medizinische Fakultät

Dekan
Prof. Dr. Dr. Uwe Koch-Gromus

Martinistraße 52
20246 Hamburg
Telefon: (040) 7410-52003
Telefax: (040) 7410-56752
dekan@uke.uni-hamburg.de
www.uke.uni-hamburg.de

Universitätsklinikum Hamburg-Eppendorf Martinistraße 52 20246 Hamburg
Dekanat der Medizinischen Fakultät

Statement of ethical considerations for the research project
"Development of admission procedures at Hamburg Medical School"

On the basis of the declaration of Helsinki „Ethical Principles for Medical Research Involving Human Subjects" (1964) we state that the study undertaken by Prof. Wolfgang Hampe conforms to the principles of medical research. We have considered the following aspects applicable to the research design (B. Principles for all medical research):

11. The study does not conflict the life, health, dignity, integrity, right to self-determination, privacy, and confidentiality of personal information of research subjects.

12. The study design is based on generally accepted scientific principles and based on thorough knowledge of the scientific literature.

13. No harm of the environment is to be expected

14. The project for the development of admission procedures is funded by the Medical School Hamburg. There are no potential conflicts of interest. Research subjects do not receive incentives for participation.

15. The research protocol is not submitted to an ethical committee. Instead, ethical considerations are undertaken by the dean of the medical school. The research protocol is according to the German laws and regulations as well as international norms and standards.

16. not applicable

17. not applicable

18. We expect benefit from the study as we gain insight into the admission process and further development of appropriate tests. The research is necessary to establish substantiated psychometric properties of the admission test. Therefore, we expect benefit to the applicants as admission procedures can be made more reliable, valid and fair. Incremental validity is an important benefit over existing admission procedures. No harm is expected for study participants.

19. not applicable

Universitätsklinikum Hamburg-Eppendorf
Körperschaft des öffentlichen Rechts
Gerichtsstand: Hamburg

Vorstandsmitglieder:
Prof. Dr. Jörg F. Debatin (Vorsitzender)
Dr. Alexander Kirstein
Joachim Prölß
Prof. Dr. Dr. Uwe Koch-Gromus

Bankverbindung:
HSH Nordbank
Kto.-Nr.: 104 364 000
BLZ: 210 500 00

20. We are confident that all risks are adequately assessed and can be managed.

21. The expected burden for the subjects is low. Since nothing is at stake and examinations are common for students, we expect participants to be able to cope with the stress which testing situations put upon some subjects. Moreover, individual benefit might result from participation as this 2-hour testing session can be considered a practice run in a science test without consequences for their further studies.

22. Study participation is voluntary and there is no disadvantage to those who do not agree to participate.

23. All data are anonymized. Only one member of the research group (not involved in teaching or examining students as they proceed in their studies) is able to decode the key in order to match test data with outcome data. After matching, all data are anonymized again for further analyses.

24. Participants are informed of all relevant aspects of the study by a consent form, including the right to refuse to participate in the study or to withdraw consent to participate at any time without reprisal.

25. Only participants who give written informed consent that their study performance data are retrieved from the data bank are considered in analyses.

26. As one of the research group members is a lecturer at the faculty, the data are edited as mentioned above (23.)

27. not applicable

28. not applicable

29. not applicable

30. All results will be published and a summary of results will be made accessible for participants on the university homepage. If participants indicate on the consent form that they would like feedback, their test scores are individually disclosed.

Prof. Dr. Dr. U. Koch-Gromus
